# GPs’ and nurses’ perceptions of electronic cigarettes in England: a qualitative interview study

**DOI:** 10.3399/bjgp18X699821

**Published:** 2018-11-06

**Authors:** Melissa Stepney, Paul Aveyard, Rachna Begh

**Affiliations:** Nuffield Department of Primary Care Health Sciences, University of Oxford, Oxford.; Nuffield Department of Primary Care Health Sciences, University of Oxford, Oxford.; Nuffield Department of Primary Care Health Sciences, University of Oxford, Oxford.

**Keywords:** electronic cigarettes, harm reduction, primary care, smoking

## Abstract

**Background:**

Reports from royal colleges and organisations such as Public Health England suggest that GPs and nurses should advise patients to switch to electronic cigarettes (e-cigarettes) if they do not want to stop smoking using licensed medication. However, there are no data on what practitioners think, feel, or do about e-cigarettes.

**Aim:**

To explore practitioners’ perceptions and attitudes towards e-cigarettes, and their experiences of discussing e-cigarettes with patients.

**Design and setting:**

A qualitative interview study was carried out with semi-structured interviews conducted with nurses and GPs across England in 2017.

**Method:**

Participants were interviewed once either via telephone or face to face. Data were analysed using thematic analysis.

**Results:**

Interviews were conducted with 23 practitioners (eight nurses and 15 GPs). There were three key themes: ambivalence and uncertainty; pragmatism; and responsibility. Many practitioners had uncertainties about the safety and long-term risks of e-cigarettes. Some had ambivalence about their own knowledge and ability to advise on their use, as well as uncertainty about whether to and what to advise patients. Despite this, many sought to provide honesty in consultations by acknowledging these uncertainties about e-cigarettes with patients and taking a pragmatic approach, believing that e-cigarettes were a ‘step in the right direction’. Practitioners wanted advice from healthcare regulators such as the National Institute for Health and Care Excellence to reassure them about the safety of e-cigarettes, practical tools to support the consultation, and to control their use by providing behavioural support programmes for reduction or cessation.

**Conclusion:**

Current dissemination strategies for guidelines are not effective in reaching practitioners, who are offering more cautious advice about e-cigarettes than guidelines suggest is reasonable.

## INTRODUCTION

Electronic cigarettes (e-cigarettes) are used by one in five people who smoke and as a cessation aid in one in three attempts to quit smoking,^1^ and the evidence is that they function much like nicotine replacement therapy to facilitate abstinence in these groups.^2^^,^^3^ Switching partially or wholly from cigarettes to e-cigarettes reduces exposure to the toxins in tobacco smoke. Guidance from Cancer Research UK, Public Health England, the Royal College of Physicians, the Royal College of General Practitioners, and the British Medical Association reflect the potential of e-cigarettes as cessation and harm reduction aids.^4^^–^7 Despite this, many people are concerned about the health harms of vaping (using e-cigarettes),^8^ with a declining minority of English smokers believing that vaping is less harmful than smoking.^1^ The guidance makes clear that GPs have a role in addressing the concerns of people who smoke and who might switch to vaping.

Survey data from the US have shown that up to one-third of smokers have asked primary healthcare providers for information and advice about e-cigarettes.^9^ Nearly two-thirds of US practitioners report being asked about e-cigarettes by patients, with around one-third recommending them as a smoking cessation tool.^9^^,^^10^ There are only a few studies on the views of primary care practitioners on e-cigarettes, with most of these conducted in the US. A common theme across these studies is that practitioners often lacked knowledge regarding the risks and benefits of e-cigarettes.^11^^,^^12^ Although they did not actively recommend their use, they did not discourage patients from using them either. Overall, practitioners wanted more empirical support on the safety and efficacy of e-cigarettes to make informed decisions on recommendations. However, in the US, official advice and regulation at the time of these studies was highly sceptical about e-cigarettes, whereas in the UK the climate and policy is different, and we might expect to see this reflected in UK practitioners’ views. The aim of this study was to explore GPs’ and nurses’ beliefs and attitudes, and reported practice on e-cigarettes.

## METHOD

The National Institute for Health Research Clinical Research Networks in Thames Valley and South Midlands, West of England, Eastern, and East Midlands regions invited registered GPs and practice nurses to participate. Guidelines suggest that both groups should be providing advice and support for people to stop smoking, including advice about vaping.^4^^–^7 Those GPs and nurses who responded were selected purposively to obtain a maximum variation sample with regard to role.

Interviews were conducted either by telephone or face to face in 2017. Informed consent was obtained orally or in writing at the beginning of the interview. GPs and nurses were reimbursed £80 and £29 per hour, respectively, following guidance from the Clinical Research Network. The topic guide was semi-structured and questions related to existing literature had been formulated from research team discussion. The topic guide covered beliefs about e-cigarettes, attitudes towards them as cessation or harm reduction aids, current practice on e-cigarette advice, views on prescribing and licensing, and the support GPs and nurses wanted in advising about e-cigarettes. Interviews lasted 35–60 minutes and were audiorecorded.

**How this fits in**

There is little information available about what practitioners think, feel, or do about electronic cigarettes (e-cigarettes) in the UK. This research provides data on practitioners’ perceptions and attitudes towards e-cigarettes, and their experiences of discussing e-cigarettes with their patients.

Interviews were transcribed and anonymised before coding in NVivo (version 10). A broad thematic analysis was carried out initially using NVivo. Each coding report was then re-analysed for further themes using mind maps to identify the key or core themes. The coding structure and core themes were discussed and agreed on with another team member. Differences and divergent cases were highlighted, as well as similarities in attitudes and experiences. Attention was also paid to how these themes related to the questions in the topic guide. Interviews continued until data saturation was reached and no new themes emerged.

## RESULTS

Forty-five GPs and practice nurses responded to the invitation letter and 23 were interviewed (15 GPs and eight nurses). The research revealed three key themes: ambivalence and uncertainty; pragmatism; and responsibility. The following section discusses GPs’ and nurses’ initial beliefs and thoughts on e-cigarettes, followed by their main uncertainties. A description of their perceptions on e-cigarettes as a device for switching and replacing smoking is followed by GPs’ and nurses’ approaches with patients and, lastly, their information needs.

### Ambivalence and uncertainty

Almost all practitioners expressed ambivalence. Although most practitioners felt that e-cigarettes were much better in terms of harms/risks than conventional cigarettes (‘the lesser of two evils’; ‘less toxins than normal cigarettes’), most believed that e-cigarettes were ‘not risk-free’. There were mixed feelings and uncertainties regarding e-cigarettes, the chief of which reflected uncertainties about the long-term effects of e-cigarettes:

‘My greater fear is that there is another harm that will become apparent over time. I think there probably will be something, just not because I’m negative, but it just doesn’t seem to me that you can inhale something for a long time without it damaging tissues.’

(GP, Thames Valley)

There were concerns about e-cigarettes triggering allergies, as well as fears that non-smokers might take up e-cigarettes: many felt they had become increasingly socially acceptable and could be taken up by non-smokers who perceived them as ‘cool’ and fashionable, especially among young people. A few practitioners also wondered about interactions between e-cigarettes and other medications, as well as alcohol and recreational drugs. One practitioner was concerned about the risks of e-cigarette nicotine to cardiovascular health. Some also talked about stories they had seen in the media. Many practitioners were uncertain that e-cigarettes supported total abstinence from smoking:

‘I suppose the idea is that e-cigarettes are likely to become a replacement rather than a weaning down to stop type therapy. Whenever we are giving nicotine replacement, we are giving it for a finite period of time as a sort of wean down to cease. I can imagine that e-cigarettes would become a chronic repeat prescription and that patients might stick with. It wouldn’t, my perception is, it might become more of a nicotine, cigarettes replacement rather than a cigarette cessation therapy and that’s the difficulty.’

(GP, West Midlands)

Several practitioners felt concerned that currently patients ‘got stuck’ on e-cigarettes, had ‘replaced one habit with another’, or had formed ‘a new addiction’. Underlying this fear was a lack of belief in harm reduction and its value:

‘So it’s sanctioning the addiction to nicotine without … working towards cessation. And, indirectly, I am also kind of drawing on my experience of prescribing methadone which is, you know, also a drug of addiction which is designed to be about replacement and weaning down and cessation. It’s really unusual getting someone off methadone. People are on it for years and you can’t help thinking, I’m not actually helping this person. I am not helping them at all. I’m writing this prescription week after week and I think it’s probably harming them and you know, maybe that again, maybe that’s the lesser evil. But it sits really uncomfortably with me. I would feel the same and again as we’ve said earlier about e-cigarettes, I don’t really know what the potential negative consequences of that drug for them are. And I am quite a cautious doctor. I don’t like prescribing … therapies for anything that haven’t been tested long term.’

(GP, Thames Valley)

In managing this ambivalence, all practitioners wanted e-cigarettes to be part of a structured programme of reduction and eventual quitting:

*‘As long as you use it* [e-cigarettes] *to stop after a few, let’s say 12 months, that’s fine. But if you are going to move from one addiction to the other, we haven’t done anything.’*

(GP, East Midlands)

Practitioners frequently described their preference for offering current treatments (which they felt more comfortable with) or referring patients on to the stop smoking service, with the ultimate aim of quitting.

### Pragmatism

Despite ambivalences and uncertainties, many practitioners took a pragmatic view. Several practitioners said that they felt that e-cigarettes were, on balance, much better and ‘a step in the right direction’. There was a sense that, despite uncertainties, practitioners weighed up the information that they knew and came to the tentative conclusion that, overall, they had a place in harm reduction:

‘In some ways I worry that it will perpetuate smoking. But on the other hand, if they are, in terms of kind of pragmatic harm minimisation, if they are safer than cigarette smoking and they are, I think, then I think they are probably a good thing.’

(GP, Thames Valley)

Even practitioners who questioned whether patients could quit smoking altogether felt, cautiously, that ‘anything’ might be better than conventional cigarettes:

*‘I think it does take them longer* [to quit on e-cigarettes]*. I think a lot of people also swap, having no intention of stopping completely. As it is safer, I encourage anything that isn’t smoking actual cigarettes. I am obviously wary about … I don’t recommend them. I encourage them to keep seeing us with the other, you know, the other help that we give advice on, dealing with cravings and things like that. I am wary because obviously not enough is known about them.’*

(Nurse, East Midlands)

Moreover, many practitioners empathised with their patients who were trying to quit. Some said quitting was a ‘process’ and that ultimately one had to be ‘realistic’ with expectations. One GP felt that e-cigarettes had ‘a place’ in making that change:

*‘I can appreciate the jump between cigarettes and not smoking is quite high, it’s quite big rather. This* [e-cigarettes] *might be a way of helping facilitating that. So it would be much more around framing it as to say, sort of step down if you like and see how you go and then we can move down beyond that, after that. Because, for some patients going cold turkey, if you like is manageable, but for many, it’s too difficult.’*

(GP, Thames Valley)

Although interviewees’ confidence levels varied when having conversations with patients (some said they were ‘reasonably confident’ in advising patients about e-cigarettes, whereas others said they were ‘unsure’ or ‘apprehensive’), many took the approach of having an ‘honest conversation’ with patients regarding their own knowledge, the long-term effects, and unknowns:

*‘And to be honest, I am very honest with them* [patients] *and say, you know, I don’t feel that it’s, in the long term, not sure about the risks with the benefits. I would say that they have not been around long enough and I don’t feel in the position to be able to fully advise them on whether the e-cigarettes are a good idea or not.’*

(Nurse, East Midlands)

‘Some of them will ask me directly, “what do you think of it?”, which is fine. And I just have exactly that conversation about, you know, about risks and unknowns with them and try and push them towards just stopping.’

(GP, Thames Valley)

Some practitioners felt that patients often knew more about e-cigarettes than they did and had already done research themselves on e-cigarettes. In this way, practitioners felt that they had a limited role and that the patient was already well informed. Almost all practitioners said that they advised their patients to go to a ‘reputable shop’ rather than buy anything on the internet.

### Responsibility

The theme of responsibility emerged strongly in many of the interviews. If practitioners were to take responsibility for prescribing or offering e-cigarettes, then they wanted a ‘higher authority’ to take responsibility for issuing recommendations. Practitioners were seeking ‘official’ sanctioning and advice about e-cigarettes (which was directed at medical professionals and not in the public domain) from one or more of the following: National Institute for Health and Clinical Excellence (NICE) guidelines; an official email/letter from Public Health England or the Royal College of General Practitioners (RCGP); and official emails or some form of guidance from the *British Medical Journal*/British Medical Association (alongside a training module). Others said that, in addition, a clinical commissioning group (CCG) prescribing circular would be useful and they wanted information about e-cigarettes on *GP Notebook*. Others mentioned that if e-cigarettes were available on prescription they would then look to the *British National Formulary* for more information:

‘*If it is recommended by NICE and then that filters to every CCG and every smoking cessation provider I’m happy to do it* [recommend e-cigarettes to patients] *but as long as it goes through the proper process … as long as it is certified and there is a process then it doesn’t matter how it comes to us.*’

(GP, East Midlands)

GPs and nurses wanted more research evidence and data on e-cigarettes to feel comfortable in advising and potentially prescribing/offering them. Specifically, they wanted more information on the following.

- The long-term potential risks of e-cigarettes: all interviewees mentioned this as the key issue they wanted to know. This was perceived as a grey area and a barrier to fully recommending them to patients.
- Data on the comparative effectiveness of e-cigarettes with current treatments like nicotine replacement therapy or varenicline.
- How e-cigarettes could be best used to facilitate total abstinence including their use in structured reduction programmes:
  ‘I would want to see evidence that e-cigarettes are a way of people stopping using nicotine rather than a replacement and how that’s best facilitated, basically. So do people need psychological support or just someone ringing them up every week or me telling them off or what is it that’s going to make it work for them?’
  (GP, Thames Valley)

### Support requested

Practitioners were keen for more information and support on e-cigarettes. This included more information and ideas about how and what to communicate with patients about e-cigarettes. They wanted ideas on how to present the uncertainty about the long-term effects. Practitioners also wanted to understand how the price of vaping compared with smoking and thought that patient decision aids showing this would be helpful. Practitioners were also keen to understand the advice they might give to subgroups of the population, such as older people, pregnant women, or young people.

Practitioners suggested that they should give patients a leaflet or booklet about e-cigarettes during consultations about smoking. Others said talking through a webpage together with a patient would be helpful. Some felt that a leaflet could be used to make a plan with the patient and identify what had been agreed (with tick boxes). Practitioners wanted this plan to cater for different approaches for heavier or lighter smokers, or even social smokers, for example. They reported that such information would in effect teach them as well as the patient about e-cigarettes:

‘I think it would be useful to have it on paper. If one had it on paper to give to patients one would also rapidly internalise it oneself, so you can have a conversation about it and then give them a bit of paper to take away.’

(GP, Thames Valley)

## DISCUSSION

### Summary

Practitioners had ambivalent views about advising and potentially prescribing e-cigarettes. On the one hand, they were ‘wary’, ‘worried’, ‘cautious’, and ‘uncertain’ about e-cigarettes. On the other hand, many weighed up the benefits of patients switching to e-cigarettes and, taking a pragmatic view, they understood this as a ‘positive step … if there’s good evidence they work’. Although they believed this, they felt cautious about expressing enthusiasm for e-cigarettes to patients because of their lack of knowledge about them and the apparent lack of official sanction for their use. As a result, practitioners often had ‘honest’ conversations emphasising the unknowns and promoting the use of licensed medication and the NHS Stop Smoking Service, in which they had more confidence. Practitioners wanted official sanction to promote e-cigarettes to know that this was the official line and that others were doing the same. They wanted practical tools to use in the consultation to promote their use.

### Strengths and limitations

GPs and nurses were sampled from across England using homogeneous purposive sampling by seeking only doctors and nurses rather than sampling on other characteristics, which were unknown to the authors. As the sampling was done by the Clinical Research Network, as is the case for most studies in England, it is impossible to know who received and declined the offer to participate. It is likely that GPs and nurses who were interested in the topic of smoking and of e-cigarettes in particular volunteered. This is likely to mean that their views were somewhat more informed on this topic than others who did not volunteer. That said, even guidance from the RCGP appears to have been unknown to most of the participants,^5^ and most were not sympathetic to ideas about harm reduction in general. Moreover, nicotine replacement therapy is currently licensed for and sometimes promoted for harm reduction, such as by cutting down on the basis of strong evidence that this leads to abstinence from smoking,^13^ and GPs and nurses appeared not to be aware of this.

In this study, although no GPs provided a cessation service themselves, seven of the eight nurses sampled provided smoking cessation services and so are likely to be more informed about smoking and smoking cessation than nurses who did not. Therefore it is likely that these interviewees, who probably had greater knowledge and interest, were more willing to take a pragmatic approach and take a degree of personal responsibility by talking with patients about e-cigarettes. The interviewer was not a health professional or an expert on tobacco control and this may have meant that practitioners were more able to express their uncertainty.

### Comparison with existing literature

Few studies have been conducted into UK healthcare providers’ knowledge and perceptions about e-cigarettes, all of which have been surveys of providers from different disciplines.^14^^–^16 Consistent with the views of GPs and nurses in this study, smoking cessation practitioners shared similar concerns about the safety of e-cigarettes and implications of their use on future dependence.^14^ As US studies found,^11^^,^^12^ most practitioners believed that e-cigarettes were a safer alternative to combustible tobacco products yet expressed some caution over actively recommending them to patients.

Previous studies show that practitioners hold similar misconceptions about licensed nicotine-containing medications and that these concerns may act as a barrier to advice giving and prescribing.^17^^,^^18^ These include fears around the long-term use of nicotine replacement therapy and concurrent use of nicotine replacement therapy and cigarettes. Similar views were expressed by GPs and nurses in this study, suggesting that primary healthcare professionals may generally lack awareness about existing guidelines^4^^–^7 on routine treatments for smoking cessation, particularly for use in harm reduction.

The findings further revealed that clinicians felt more comfortable advising on e-cigarette use within a structured programme because this reduced their fears about transferring dependence or anxiety about the value of harm reduction. Although this is understandable, it may not be desirable from the public health perspective. Healthcare practitioners have spent more than a decade promoting the use of the NHS Stop Smoking Service and the use of licensed medication, with modest success in terms of numbers of users.^1^ This is despite the fact that behavioural support and medication are available at modest or no cost for users.

E-cigarettes were initially launched with little promotion and modest financial backing, and use of these products has eclipsed the use of officially sanctioned routes to quit several-fold, despite these products entailing greater costs for users.^19^ Arguably, therefore, it is the lack of official sanction that may be part of the appeal of e-cigarettes to users and seeking to control their use through cessation or harm reduction programmes may undermine their attractiveness and use.

### Implications for practice and policy

These results affirm what many believe to be true, which is that guidelines alone rarely change practice.^20^ Few practitioners had come across these and made deductions about e-cigarette use largely drawn from common sense. However, practitioners’ strong sense of professional responsibility meant that they tended to give more cautious advice on e-cigarettes than the existing guidelines suggest is reasonable.^5^^–^7 This suggests that active dissemination of these guidelines is required. However, public health authorities should feel consoled that practitioners actively want this kind of support. Moreover, practitioners have clear requests for support materials that may improve implementation to support dissemination.

Perhaps surprisingly, these practitioners expressed willingness to have discussions with patients about smoking and stopping smoking. Previous research has shown that practitioners express a lack of enthusiasm about these conversations,^21^^,^^22^ but it is clear from this study that practitioners want support to respond to requests from patients.
